# Predictive value of aneurysm characteristics for one-year persistence in Kawasaki disease: a retrospective cohort study

**DOI:** 10.3389/fcvm.2025.1733982

**Published:** 2025-12-19

**Authors:** Shang Lifeng, Su Danyan, Qin Suyuan, Chen Cheng, Qiao Xiaoyu, Sun Lu, Wang Zhouping, Pang Yusheng

**Affiliations:** 1 Difficult and Critical Illness Center and the Pediatric Clinical Medical Research Center of Guangxi, The First Affiliated Hospital of Guangxi Medical University, Nanning, Guangxi, China; 2 Heart Center and Institute of Pediatrics, Guangzhou Women and Children’s Medical Center, Guangzhou Medical University, Guangdong Provincial Clinical Research Center for Child Health, Guangzhou, China

**Keywords:** aneurysm location, aneurysm morphology, coronary artery aneurysms, Kawasaki disease, *Z*-score

## Abstract

**Objective:**

While systemic factors influence Kawasaki disease outcomes, this study specifically determines the independent and incremental prognostic value of the coronary aneurysm's own characteristics—maximum *Z*-score (ZM), morphology, and Location—for predicting persistence one year after onset.

**Methods:**

This retrospective cohort study enrolled 135 children with KD and coronary artery aneurysms (CAA). We analyzed the maximum *Z*-score (ZM), morphology (fusiform/saccular), and coronary artery Location (Left/Right/Bilateral) of the index aneurysm (the largest by *Z*-score). Univariable and multivariable logistic regression were used to identify independent predictors. The predictive performance of a model containing only ZM was compared to that of the Comprehensive Aneurysm Characteristic (CAC) model, which incorporates ZM, morphology, and Location, by assessing the area under the receiver operating characteristic curve (AUC). A descriptive analysis was additionally performed on a high-risk subgroup defined by a ZM ≥ 5.0.

**Results:**

The early ZM was a strong predictor of persistent coronary aneurysms at one year (OR = 4.925, *P* < 0.001), with an AUC of 0.909. In the multivariable analysis, larger ZM (aOR = 6.775, 95% CI: 3.133–14.648, *P* < 0.001), saccular morphology (aOR = 7.648, 95% CI: 1.428–40.967, *P* < 0.05), and LAD involvement (aOR = 4.304, 95% CI: 1.163–15.928, *P* < 0.05) emerged as independent predictors. The CAC model demonstrated a statistically significant improvement in predictive ability over the ZM-only model (AUC: 0.941 vs. 0.909, *P* = 0.025). Exploratory analysis of the high-risk subgroup (ZM ≥ 5.0) revealed a markedly higher prevalence of saccular morphology in patients with persistent aneurysms, suggesting it may serve as a crucial risk signal independent of absolute size in this population. The CAC model also showed excellent calibration and superior clinical utility across a wide range of decision thresholds.

**Conclusion:**

The intrinsic characteristics of a coronary aneurysm—its size, shape, and distribution—collectively provide powerful, independent prediction of its persistence at one year. The strong association of specific morphological features with persistence in the high-risk subgroup underscores the value of anatomy-based assessment for refining risk stratification, complementing evaluations based on systemic host factors.

## 1 Background

Kawasaki disease (KD) is one of the most common acquired heart diseases in childhood (1). Its most serious complication is the formation of coronary artery aneurysms (CAA) (2). Currently, the initial aneurysm size, typically measured by the maximum *Z*-score (ZM), serves as a fundamental predictor for its persistence in clinical practice (3, 4). However, relying solely on size for prediction has limitations (5). Clinical observations indicate that even aneurysms with similar initial sizes can have significantly different outcomes (6). This uncertainty in prediction based on a single feature is particularly prominent in the large patient population with small- to medium-sized aneurysms, highlighting the future need for multi-omics research and standardized monitoring to improve individualized management (7). While systemic factors such as age (8), inflammatory burden (9), and genetic susceptibility (10) contribute to this heterogeneity, we posit that the intrinsic imaging characteristics of the aneurysm itself—the direct morphological sequela of the vascular injury-may harbor decisive prognostic information. Specifically, beyond absolute size, the morphology (saccular vs. fusiform) and pattern of coronary involvement (location) may reflect the severity of local wall disruption and the extensiveness of the pan-coronary insult, respectively.

Nevertheless, the combined and independent prognostic value of these three key aneurysm-centric features—size, morphology, and Location—for predicting one-year persistence has not been systematically evaluated within a unified model. Such an anatomy-focused assessment could provide a crucial, complementary dimension to prediction models based primarily on systemic host factors.

Therefore, this study aimed to develop and validate a Comprehensive Aneurysm Characteristic (CAC) model to determine whether the integration of aneurysm size (ZM), morphology, and location provides incremental predictive value over size alone for predicting one-year persistence of coronary aneurysms in KD.

## 2 Methods

### 2.1 Study design and participants

This retrospective cohort study utilized clinical data from pediatric patients diagnosed with KD complicated by CAA at Guangzhou Women and Children's Medical Center between January 2015 and December 2023. During this period, a total of 140 patients were diagnosed with KD and CAA. The inclusion criteria were: (1) meeting the established diagnostic criteria for KD; (2) age ≤18 years at diagnosis; (3) echocardiographically confirmed CAA during the acute phase, defined as a maximum coronary artery *Z*-score ≥2.5 in any segment; and (4) availability of complete serial echocardiographic data from both the acute phase (within 1 month of onset) and at the 1-year follow-up. Exclusion criteria included: (1) significant missing clinical data (*n* = 0), and (2) lost to follow-up or incomplete 1-year follow-up data (*n* = 5). Ultimately, 135 patients met all criteria and were included in the final analysis. The study design and statistical analysis were conducted collaboratively by investigators from Guangzhou Women and Children's Medical Center and The First Affiliated Hospital of Guangxi Medical University.

### 2.2 Data collection and quality control

To ensure consistent and objective assessments, a rigorous, multi-step adjudication process was implemented for data collection. Two experienced cardiovascular ultrasound specialists, who were blinded to all patient outcomes and clinical data, independently conducted a retrospective review of all stored echocardiographic images and reports from the study period (January 2015 to December 2023). They recalculated coronary artery *Z*-scores using the 2017 American Heart Association (AHA) formulas. Aneurysm morphology was classified as fusiform (longitudinal dimension > transverse dimension) or saccular (transverse dimension ≥ longitudinal dimension). Any discrepant cases in morphological classification were adjudicated by a third senior specialist. The final morphology and *Z*-score for each patient were determined by consensus among all three experts. For patients with aneurysms in multiple locations, the lesion with the largest *Z*-score was designated as the index aneurysm for analysis. Based on this consensus data, the maximum *Z*-score (ZM) during the early disease phase was designated as the primary predictor. Baseline demographic characteristics, clinical features, and laboratory parameters were collected from routine medical records.

### 2.3 Predictor and outcome definitions

Index Aneurysm: For patients with multiple aneurysms, the single aneurysm with the highest *Z*-score during the acute phase was designated as the “index aneurysm” for all subsequent analyses. This approach ensured one data point per patient and focused on the most severe lesion. Predictor Variables: Size: The maximum *Z*-score of the index aneurysm (ZM), treated as a continuous variable. Morphology: The shape of the index aneurysm, classified as fusiform (longitudinal dimension > transverse dimension) or saccular (transverse dimension ≥ longitudinal dimension). Morphology was assessed by reviewing stored echocardiographic images and reports. Location: Aneurysms located in the left anterior descending artery or the right coronary artery. Outcome Definition: The primary outcome was the persistence of the index aneurysm at the 1-year follow-up (±1 month), defined as the index aneurysm maintaining a *Z*-score ≥2.5.

### 2.4 Definition

IVIG non-response was defined as recurrence of fever (axillary temperature ≥38.0 °C) at least 36 h after the completion of the initial IVIG (2 g/kg) infusion, accompanied by persistent or recrudescent clinical signs of KD. Delayed IVIG treatment was defined as the initiation of IVIG therapy later than 10 days from the onset of fever.

### 2.5 Treatment and follow-up

According to medical records, all patients with coronary artery aneurysms received antithrombotic therapy based on aneurysm severity. For patients with small aneurysms (ZM: 2.5–5.0), the initial recorded regimen was aspirin. For children with concomitant G6PD deficiency, the recorded treatment was switched to clopidogrel. For patients with medium aneurysms (ZM: 5.0–10.0), records indicated that most received aspirin monotherapy (*n* = 15). However, 8 patients (34.8%) with high-risk features (e.g., multiple aneurysms, saccular morphology) received combined therapy with aspirin and clopidogrel, as detailed in Table 5. For the patient with a large aneurysm (ZM ≥ 10.0), the medical record indicated treatment with aspirin, clopidogrel, and warfarin. The primary outcome of this study was anatomical persistence of the aneurysm (*Z*-score ≥2.5). Additionally, the occurrence of Major Adverse Cardiovascular Events (MACE), defined as a composite of myocardial infarction, coronary intervention, or cardiac death, as well as any coronary thrombus formation, was retrospectively reviewed during the one-year follow-up period.

### 2.6 Statistical analysis

Continuous variables were tested for normality using the Shapiro–Wilk test. Normally distributed data are presented as mean ± standard deviation and compared using independent samples *t*-tests, while non-normally distributed data are expressed as median (interquartile range) and compared using Mann–Whitney *U* tests. Categorical variables are summarized as counts (percentages) and compared using chi-square or Fisher's exact tests as appropriate. Univariate and multivariate logistic regression models were employed to assess independent effects. The predictive performance of the maximum *Z*-score was evaluated using receiver operating characteristic (ROC) curve analysis. The overall performance of the final CAC model was further assessed by evaluating calibration and clinical utility. A univariate logistic regression analysis was performed on a comprehensive set of baseline characteristics to screen for associations with the primary outcome; the full results are presented in Supplementary Table S1. It should be noted that all reported performance metrics for the CAC model represent “apparent” performance estimates derived from the development dataset, which are likely optimistic and represent a best-case scenario; internal validation using bootstrapping was not performed due to resource constraints and sample size considerations; thus external validation is required to confirm generalizable performance. All analyses were performed using SPSS 26.0 and R 4.2.0, with a two-sided *p*-value <0.05 considered statistically significant.

Given the limited number of outcome events and to prevent overfitting of the multivariable model, we pre-specified a parsimonious set of predictors based on the study's primary aim. The core predictors were the intrinsic aneurysm characteristics: maximum *Z*-score (ZM), morphology, and location. Although univariate analysis identified several systemic and clinical factors associated with the outcome (Supplementary Table S1), these were not included in the primary multivariable model. This approach ensures model stability and focuses the analysis on evaluating the incremental value of aneurysm anatomy beyond size alone. The role of systemic factors is acknowledged and discussed in the context of the literature.

### 2.7 Ethics

In accordance with the Declaration of Helsinki, institutional review board (IRB) approval was sought. The IRB granted exemption from ethical review for this study. As data were anonymized, the requirement for informed consent was waived.

## 3 Results

### 3.1 Baseline characteristics

A total of 135 children with Kawasaki disease and coronary artery aneurysms (CAA) were included in the final analysis. Based on the CAA status at the 1-year follow-up, patients were stratified into two groups: the CAA regression group (*n* = 105) and the CAA persistence group (*n* = 30). The CAA persistence group demonstrated a significantly higher median ZM during the early phase compared to the regression group (5.69 vs. 3.19, *P* < 0.001). A comprehensive comparison of all baseline clinical and demographic characteristics between the two groups is detailed in Table 1. Significant intergroup differences (*P* < 0.05) were also observed in multiple baseline parameters, including age, height, total fever duration, laboratory findings (white blood cell count, platelet count, absolute neutrophil count, NT-proBNP, chloride, total protein, globulin, albumin-to-globulin ratio), and the proportion of patients who received delayed intravenous immunoglobulin treatment. These baseline differences suggest that children in the CAA persistence group may have experienced a more severe inflammatory response and overall disease severity during the acute phase, which is consistent with previous research. As this study primarily aims to investigate the predictive value of the aneurysms' own imaging characteristics, the subsequent analysis focused on the three core factors: ZM, morphology, and location. The results of the univariate analysis for all baseline characteristics are provided in Supplementary Table S1.

**Table 1:** Baseline clinical and demographic characteristics of the study cohort.

Variables	CAA regression (*N* = 105)	CAA persistence (*N* = 30)	*P*
Gender			0.764
Female	23 (21.90%)	8 (26.67%)	
Male	82 (78.10%)	22 (73.33%)	
Resistan			0.36
No	90 (85.71%)	28 (93.33%)	
Yes	15 (14.29%)	2 (6.67%)	
Age (month)	18.00 [8.00; 28.00]	28.00 [10.25; 45.00]	0.046*
Kg	10.50 [8.50; 12.50]	12.45 [8.85; 16.45]	0.075
M	0.81 [0.70; 0.90]	0.88 [0.74; 1.06]	0.028*
Incomplete			0.688
No	66 (62.86%)	17 (56.67%)	
Yes	39 (37.14%)	13 (43.33%)	
Total duration of fever (day)	8.00 [7.00; 11.00]	12.00 [8.00; 15.00]	0.004**
White blood cell (×10^9^/L)	18.38 [16.01; 20.39]	20.57 [18.31; 23.25]	0.016*
Red blood cell (×10^12^/L)	4.06 [3.69; 4.43]	4.20 [3.83; 4.54]	0.347
Hemoglobin (g/L)	100.32 (12.11)	102.54 (11.47)	0.36
Platelet (×10^9^/L)	395.00 [311.00; 546.00]	469.50 [402.50; 658.25]	0.007**
Neutrophils (×10^9^/L)	12.29 [9.79; 15.23]	14.18 [12.63; 16.51]	0.038*
Neutrophils (%)	67.63 (12.96)	70.82 (12.43)	0.225
CRP (mg/L)	107.05 [85.59; 135.31]	115.72 [98.75; 132.12]	0.525
PCT (ng/mL)	1.20 [0.36; 2.67]	1.53 [0.23; 4.13]	0.743
ESR (mm/h)	50.00 [34.00; 75.00]	53.00 [30.25; 84.25]	0.481
NT_proBNP (pg/mL)	715.00 [255.32; 2054.59]	312.88 [143.97; 583.46]	0.046*
K^+^ (mmol/L)	3.85 (0.55)	3.73 (0.53)	0.285
Na^+^ (mmol/L)	136.00 [135.00; 138.00]	137.05 [134.75; 138.33]	0.44
Cl^−^ (mmol/L)	99.40 [97.90; 101.80]	98.20 [96.08; 99.23]	0.003**
Ca^2+^ (mmol/L)	2.27 [2.14; 2.36]	2.30 [2.20; 2.35]	0.536
Total Bilirubin (umol/L)	4.60 [3.20; 7.10]	4.95 [2.95; 9.90]	0.641
Direct bilirubin (umol/L)	2.00 [1.20; 3.60]	2.20 [1.35; 5.08]	0.733
Indirect bilirubin (umol/L)	2.70 [1.60; 3.70]	2.45 [1.70; 4.47]	0.608
Total Protein (g/L)	63.76 (7.75)	67.00 (6.64)	0.027*
Albumin (ALB, g/L)	34.60 [31.40; 37.00]	35.45 [31.08; 37.18]	0.759
Globulin (Glob, g/L)	27.50 [24.50; 33.00]	31.95 [27.85; 34.80]	0.009**
Alb/Glob	1.22 (0.35)	1.09 (0.28)	0.041*
*γ*-Glutamyl transpeptidase (γ-GT, U/L)	58.00 [26.00; 123.00]	58.00 [23.00; 99.25]	0.387
Total bile acids (umol/L)	7.98 [4.10; 15.80]	8.85 [5.12; 21.88]	0.35
Aspartate aminotransferase (U/I)	28.00 [22.00; 38.00]	23.00 [21.25; 31.50]	0.07
Alanine aminotransferase (U/I)	32.00 [18.00; 70.00]	22.00 [12.25; 58.75]	0.071
Alkaline phosphatase (U/L)	179.00 [135.00; 221.00]	183.50 [135.50; 220.75]	0.895
Lipase (U/L)	36.00 [28.00; 47.00]	37.50 [31.25; 59.75]	0.186
Urea nitrogen (mmol/L)	3.00 [2.40; 3.60]	3.27 [2.91; 3.61]	0.161
Creatinine (umol/L)	20.00 [17.00; 25.00]	23.00 [18.00; 29.00]	0.054
Uric acid (umol/L)	185.00 [151.00; 233.00]	200.50 [163.75; 256.25]	0.222
Lactate (mmol/L)	2.00 [1.60; 2.60]	2.10 [1.83; 2.48]	0.392
CystatinC (umol/L)	0.83 [0.75; 0.95]	0.83 [0.74; 0.93]	0.645
Creatine kinase (CK,U/L)	31.00 [19.00; 48.00]	27.50 [16.00; 42.75]	0.436
Creatine kinase-myocardial band (CKMB,U/L)	14.00 [10.00; 19.00]	13.00 [11.25; 20.25]	0.844
CK/CKMB	0.48 [0.27; 0.72]	0.48 [0.27; 0.78]	0.859
Lactate dehydrogenase (U/L)	259.00 [232.00; 293.00]	245.50 [218.00; 266.75]	0.076
Thrombin activity (%)	91.00 [83.00; 102.00]	95.00 [82.50; 108.00]	0.343
Activated partial thromboplastin time (s)	39.70 [36.00; 44.20]	42.80 [37.40; 45.32]	0.087
Prothrombin time (s)	13.80 [13.10; 14.40]	13.60 [13.00; 14.50]	0.628
Fibrinogen (g/L)	6.07 (1.22)	6.48 (1.27)	0.118
Thrombin time (s)	16.76 (1.64)	16.42 (1.62)	0.319
INR	1.06 [0.99; 1.11]	1.04 [0.99; 1.13]	0.874
IVIG delay			0.002**
No	84 (80.00%)	15 (50.00%)	
Yes	21 (20.00%)	15 (50.00%)	
Maximum *Z*-score (ZM)	3.19 [2.69; 4.11]	5.69 [4.85; 6.29]	<0.001***

* *P* < 0.05.

** *P* < 0.01.

*** *P* < 0.001.

### 3.2 Predictive performance of maximum *Z*-score

**Figure 1:**
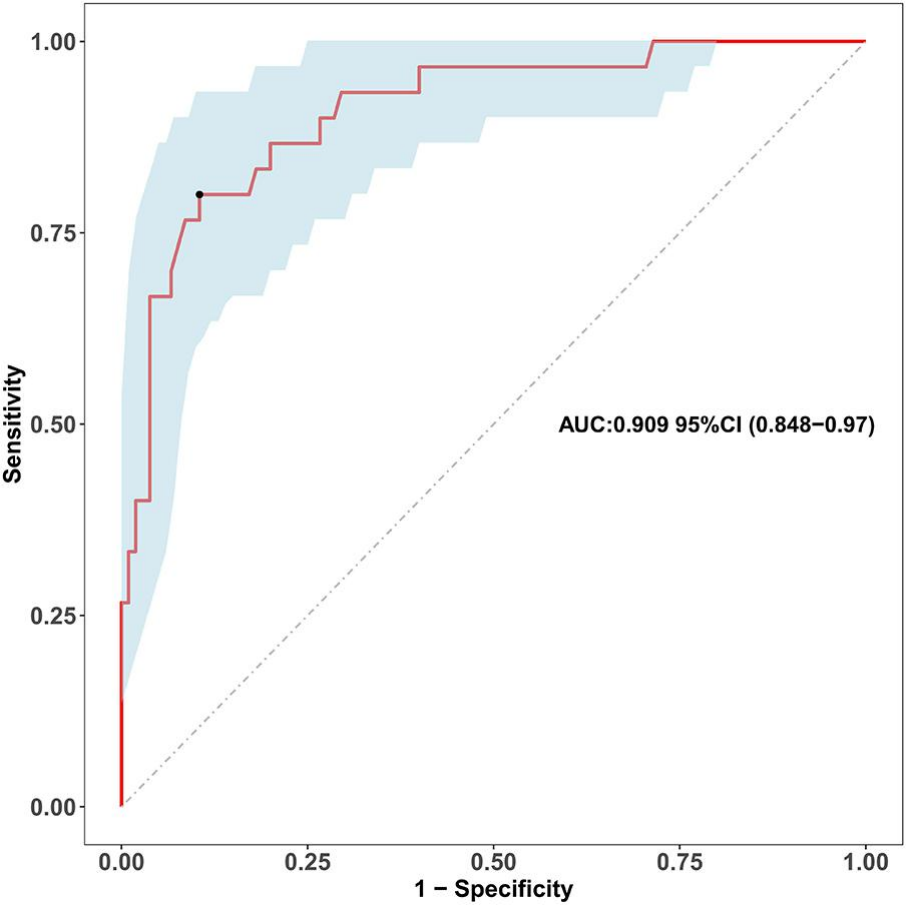
Receiver operating characteristic (ROC) curve of the maximum early-term coronary *Z*-score for predicting coronary aneurysm persistence. The receiver operating characteristic (ROC) curve evaluates the discriminatory ability of the maximum coronary artery *Z*-score within the first month of illness (as a continuous variable) for predicting persistent coronary aneurysms at the 1-year follow-up. The area under the curve (AUC) is 0.909 (95% confidence interval: 0.848-0.970). The diagonal dashed line represents the reference line of no discrimination.

Univariable logistic regression identified the ZM within the first month as a powerful predictor of persistent coronary aneurysms at 1 year (OR = 4.925, 95% CI: 2.738–8.856, *P* < 0.001). Receiver operating characteristic (ROC) analysis demonstrated excellent discriminatory power with an area under the curve (AUC) of 0.909 (95% CI: 0.848–0.970; Figure 1).

### 3.3 Aneurysm characteristics and multivariable prediction model

The baseline distribution of the key aneurysm characteristics is summarized in Table 2. Saccular morphology was present in 20.7% of the cohort, while LAD involvement was observed in 41.5% of patients. Both characteristics demonstrated a strong bivariate association with the outcome, being markedly more prevalent in the persistence group (46.7% and 60.0%, respectively) than in the regression group (13.3% and 36.2%, *P* < 0.001 for morphology, *P* = 0.034 for location). Furthermore, a larger initial aneurysm size, as measured by the ZM, was a prominent feature in the persistence group (Median: 5.69, IQR: 4.85–6.29) compared to the regression group (Median: 3.19, IQR: 2.69–4.11, *P* < 0.001).

**Table 2:** Baseline characteristics of index aneurysm characteristics.

Variables	Total (*n* = 135)	CAA regression (*n* = 105)	CAA persistence (*n* = 30)	*P*
Morphology *n* (%)				<0.001***
Fusiform	107 (79.26)	91 (86.67)	16 (53.33)	
Saccular	28 (20.74)	14 (13.33)	14 (46.67)	
Location, *n* (%)				0.034
RCA	79 (58.52)	67 (63.81)	12 (40.00)	
LAD	56 (41.48)	38 (36.19)	18 (60.00)	
ZM, Median (Q1,Q3)	3.5 (2.76, 4.74)	3.19 (2.69, 4.11)	5.69 (4.85, 6.29)	<0.001***

Comparison of aneurysm size (ZM), morphology, and location between patients with and without aneurysm persistence at one year. Data are presented as *n* (%) or median (IQR). *P*-values are from Chi-squared tests (morphology, location) and Mann–Whitney *U* test (ZM).

*** *P* < 0.001.

The results of the univariable and multivariable logistic regression analyses are presented in Table 3. In univariable analysis, larger ZM, saccular morphology, and LAD involvement were all significantly associated with aneurysm persistence (all *P* < 0.05). In the multivariable model that included all three characteristics, larger ZM (adjusted OR = 6.775, 95% CI: 3.133–14.648, *P* < 0.001) and LAD involvement (adjusted OR = 4.304, 95% CI: 1.163–15.928, *P* < 0.05) emerged as independent predictors with precise effect estimates.

**Table 3:** Aneurysm characteristics as predictors of one-year persistence: univariate and multivariable analyses.

Characteristics	Uni *β*	Uni SE	Uni OR 95% CI	Uni *P*	Multi *β*	Multi SE	Multi OR 95% CI	Multi *P*
Morphology	1.738	0.46513	5.688 (2.297–14.39)	<0.001***	2.034	0.85628	7.648 (1.428–40.967)	<0.05*
Location	0.973	0.42442	2.645 (1.162–6.208)	<0.05*	1.46	0.66757	4.304 (1.163–15.928)	<0.05*
ZM	1.594	0.29941	4.925 (2.921–9.592)	<0.001***	1.913	0.39343	6.775 (3.133–14.648)	<0.001***

Uni, univariate; Multi, multivariable; *β*, regression coefficient; SE, standard error; OR, odds ratio; CI, confidence interval; ZM, maximum *Z*-score. The CVC model, adjusted for all three characteristics, identifies larger ZM and saccular morphology as independent predictors of aneurysm persistence. Location was significant in univariate analysis and in the adjusted model.

* *P* < 0.05.

*** *P* < 0.001.

Saccular morphology also demonstrated a significant association after adjustment (adjusted OR = 7.648, 95% CI: 1.428–40.967, *P* < 0.05). However, the considerable widening of its confidence interval indicates instability in the point estimate, likely reflecting limited statistical power within this subgroup. Therefore, while these results support its role as an independent risk factor, the exact magnitude of its effect remains uncertain. Given this statistical imprecision alongside its strong unadjusted association, we further explored its clinical relevance in the high-risk subgroup analysis below.

**Figure 2:**
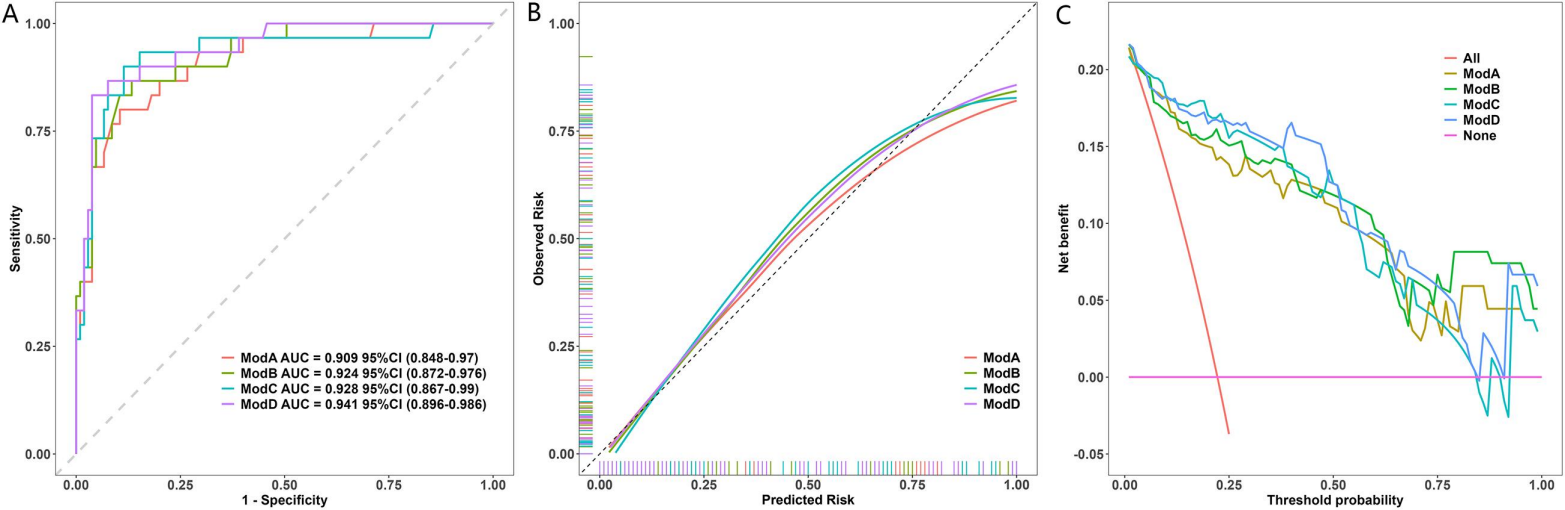
Performance of the Model. **(A)** ROC curves of the CAC model vs. the ZM-only model. AUC values with 95% CIs are shown. The difference was significant (DeLong's test, *P* = 0.025). **(B)** Calibration plot of the CAC model. The dashed line represents the ideal fit. The Hosmer-Lemeshow test indicated excellent fit (*P* = 0.786). **(C)** Decision curve analysis. The CAC model provided higher net benefit than “All” or “None” strategies across most thresholds.

The results of the model performance assessments are presented in Figure 2. The discriminatory ability of the four models was evaluated using ROC analysis (Figure 2A). The model based solely on the maximum *Z*-score (ModA) served as the baseline, achieving an AUC of 0.909 (95% CI: 0.848–0.970). The multivariable models, which incorporated additional aneurysm characteristics, demonstrated progressively higher AUC values: the model combining ZM and morphology (ModB) achieved an AUC of 0.924 (95% CI: 0.872–0.976); the model combining ZM and location (ModC) achieved an AUC of 0.928 (95% CI: 0.867–0.990); and the comprehensive model incorporating ZM, morphology, and location (ModD) achieved the highest AUC of 0.941 (95% CI: 0.896–0.986). DeLong's test for correlated ROC curves indicated that the superiority of the comprehensive model (ModD) over the baseline ModA was statistically significant (difference in AUC = 0.032, 95% CI for difference: 0.004–0.060, *P* = 0.025). In contrast, the improvements of ModB (difference in AUC = 0.015, *P* = 0.347) and ModC (difference in AUC = 0.019, *P* = 0.118) over ModA were not statistically significant. The calibration curve (Figure 2B) showed that the predicted probabilities of the primary model were in close agreement with the observed outcomes across the entire range of risk. Decision curve analysis (Figure 2C) was employed to evaluate clinical utility. All four models provided a greater net benefit than the “treat all” or “treat none” strategies across a wide range of clinically reasonable threshold probabilities. Among them, the comprehensive model (ModD) yielded the highest net benefit, solidifying its value for informing clinical decisions.

### 3.4 Temporal evolution of ZM

**Figure 3:**
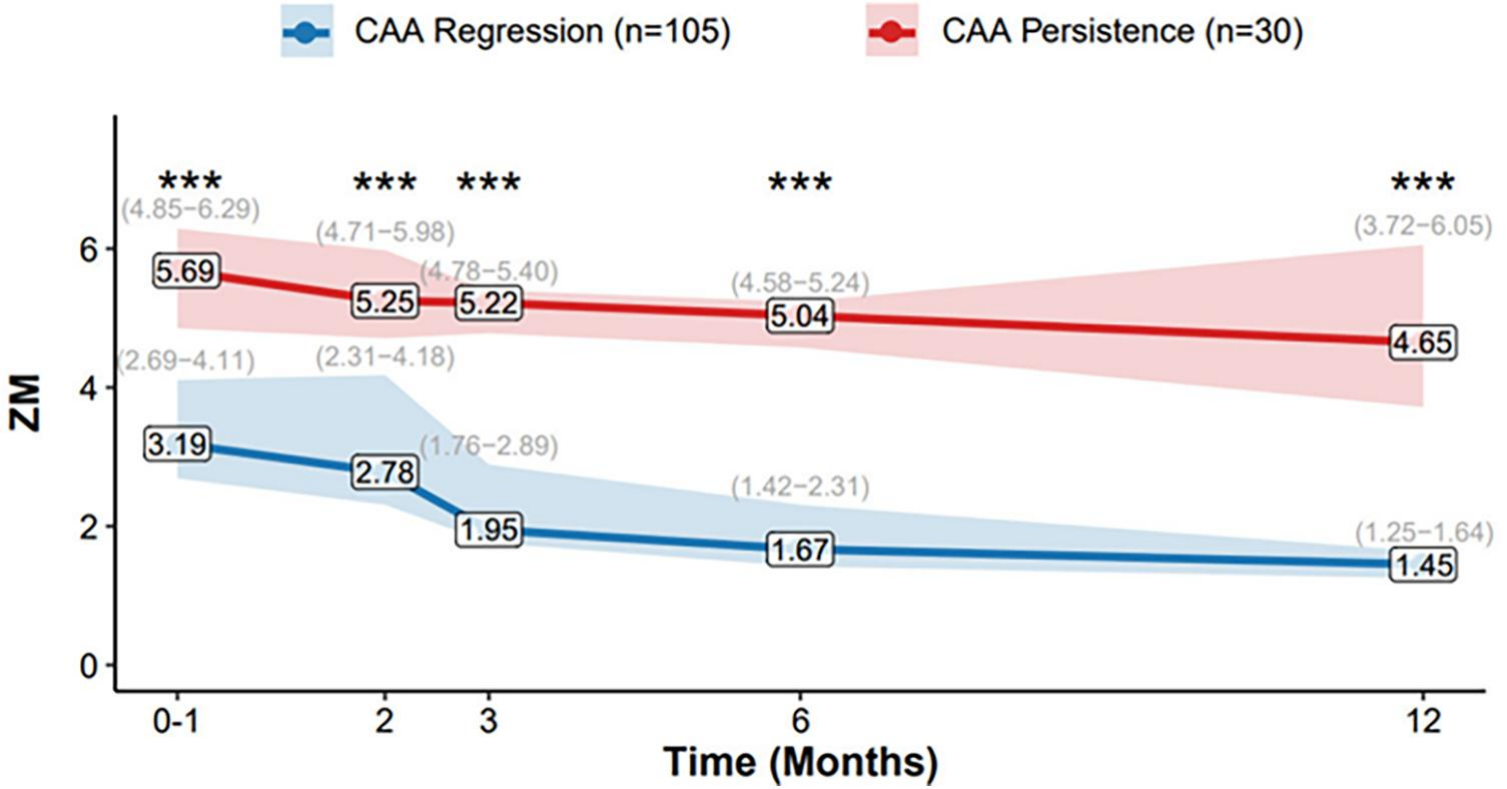
Temporal Evolution of ZM. Data presented as median (IQR: Q25−Q75), lines connect the median values at each time point (0–1, 2, 3, 6, and 12 months). The shaded areas represent the interquartile range (IQR, from 25th to 75th percentile). Group sample sizes are *n* = 30 (CAA Persistence) and *n* = 105 (CAA Regression) at all time points. *p < 0.05, **p < 0.01, ***p < 0.001.

The longitudinal evolution of ZM from the acute phase to 1-year follow-up is summarized in Figure 3. Patients with CAA regression demonstrated a pronounced downward trend in median *Z*-scores over time, with values decreasing from 3.19 (IQR: 2.65–4.23) at baseline to 1.42 (IQR: 1.23–1.54) at 12 months. In contrast, the CAA persistence group maintained elevated *Z*-scores throughout the follow-up period, with median values of 5.69 (IQR: 4.86–6.88) at baseline and 4.80 (IQR: 3.69–5.42) at 12 months.

### 3.5 High-risk subgroup analysis

We conducted a descriptive analysis on the high-risk subgroup of patients with a maximum *Z*-score (Zmax) ≥ 5.0 (*n* = 24). Owing to the limited sample size of this subgroup (*n* = 24), conducting a stable multivariable analysis was not feasible. The following findings should therefore be interpreted as exploratory and hypothesis-generating. The baseline characteristics and aneurysm features of this subgroup, stratified by persistence outcome at one year, are detailed in Table 4. Within this high-risk cohort, 10 patients (41.7%) had persistent aneurysms. The persistence group had a significantly larger initial aneurysm size (Median Zmax: 7.46 vs. 5.76, *P* < 0.001). Consistent with the signal of elevated risk observed in the primary multivariable model, saccular morphology was overwhelmingly more prevalent in the persistence group (90.0%) compared to the regression group (14.3%, *P* = 0.001). Involvement of the left anterior descending artery (LAD) was also more common in the persistence group (70.0% vs. 35.7%), although this difference did not reach statistical significance (*P* = 0.214). No other baseline clinical characteristics showed significant differences between the groups.

**Table 4:** Characteristics and aneurysm features of the high-risk subgroup (Zmax ≥ 5.0) stratified by one-year persistence.

Variables	Aneurysm regression (*N* = 14)	Aneurysm persistence (*N* = 10)	*P*
Demographics			
Male Gender, *n* (%)	10 (71.4)	7 (70.0)	1
Age (months), Median [IQR]	8.5 [5.3; 21.8]	15.5 [8.5; 51.8]	0.218
Clinical Features			
IVIG Resistance, *n* (%)	2 (14.3)	2 (20.0)	1
Incomplete KD, *n* (%)	6 (42.9)	6 (60.0)	0.679
Total Duration of Fever (days), Mean (SD)	11.4 (5.4)	12.9 (3.8)	0.422
Delayed IVIG Treatment, *n* (%)	7 (50.0)	6 (60.0)	0.697
Index Aneurysm Characteristics			
Maximum *Z*-score (Zmax), Median [IQR]	5.76 [5.68; 5.88]	7.46 [6.38; 8.22]	<0.001***
Saccular Morphology, *n* (%)	2 (14.3)	9 (90.0)	0.001**
LAD Involvement, *n* (%)	5 (35.7)	7 (70.0)	0.214

This table presents a descriptive analysis of the high-risk subgroup. Data are presented as *n* (%), mean (standard deviation), or median [interquartile range]. *P*-values are provided for context but should be interpreted with caution due to the small sample size (*n* = 24) of this exploratory analysis. Comparisons were made using Fisher's exact test (for categorical variables with expected counts <5), Chi-squared tests, independent samples *t*-tests, or Mann–Whitney *U* tests, as appropriate. IVIG, intravenous immunoglobulin; KD, Kawasaki disease; LAD, left anterior descending artery.

**P < 0.01.

*** *P* < 0.001.

### 3.6 Clinical events and pharmacotherapy details

The antithrombotic regimens for the entire cohort are summarized in Table 5. In brief, among the 111 patients with small aneurysms, 12 with G6PD deficiency received clopidogrel instead of aspirin. Of the 23 patients with medium aneurysms, 8 received dual antiplatelet therapy with aspirin and clopidogrel. The single patient with a giant aneurysm was on a triple therapy regimen of aspirin, clopidogrel, and warfarin.

**Table 5:** Antiplatelet and anticoagulant therapy.

Aneurysm Grade	Number of Patients (n)	Recorded Antithrombotic Regimen
Small CAA	111	Aspirin (*n* = 99); Clopidogrel (*n* = 12, for G6PD deficiency)
Medium CAA	23	Aspirin (*n* = 15); Aspirin + Clopidogrel (*n* = 8)
Large CAA	1	Aspirin + Clopidogrel + Warfarin (*n* = 1)

No Major Adverse Cardiovascular Events (MACE) were documented during the follow-up period. However, coronary thrombus formation was identified in 2 patients. The detailed clinical characteristics and management of these thrombosis cases are presented in Table 6. Both patients were treated with a protocol of urokinase thrombolysis followed by a heparin sodium bridge, culminating in long-term warfarin therapy. Medical records indicated that the thrombi were controlled in both instances following these interventions.

**Table 6:** Details of coronary thrombosis cases.

Case	CAA Size	Medication Prior to Thrombosis	Management Measures
1	Large CAA	Aspirin + Clopidogrel + Warfarin	Urokinase thrombolysis → Heparin sodium bridge → Long-term Warfarin
2	Medium CAA	Aspirin + Clopidogrel	Urokinase thrombolysis → Heparin sodium bridge → Initiation of long-term Warfarin

## 4 Discussion

This study developed and validated a comprehensive aneurysm characteristic (CAC) model demonstrating that integrating aneurysm size, morphology, and location provides a significantly superior tool for predicting one-year persistence. The CAC model showed statistically significant improvement in discrimination over the size-only model (AUC: 0.941 vs. 0.909, *P* = 0.025), along with excellent calibration and greater net clinical benefit, offering a robust, anatomy-based approach for individualized risk stratification in children with Kawasaki disease and coronary artery aneurysms.

Our findings first reaffirm the established role of initial aneurysm size, measured by the maximum *Z*-score (ZM), as a cornerstone for risk prediction (3). The high discriminatory power of early ZM (AUC = 0.909) aligns with current guideline recommendations. However, the significant outcome heterogeneity observed among patients with aneurysms of similar initial size underscores the limitation of relying on a single dimension (6), coupled with the recognized high regression rate of small- to medium-sized aneurysms (*Z*-score 2.5–1.0) (11–13), collectively underscores the limitation of relying on a single dimension and highlights the necessity of incorporating additional anatomical characteristics for refined prognostication. While predicting persistence in the broader cohort (*Z*-score ≥ 2.5) establishes the general principle of the CAC model's utility, we acknowledge that the clinical imperative is greatest for larger aneurysms (*Z*-score ≥ 5.0). Our subsequent analysis therefore focused on this high-risk subgroup to address this priority directly.

To directly address the clinical relevance of predicting outcomes in larger aneurysms, we conducted a descriptive analysis of the high-risk subgroup with ZM ≥ 5.0. Within this cohort, our exploratory analysis revealed that saccular morphology and LAD involvement were strikingly more prevalent in patients with persistent aneurysms. Specifically, the proportion of saccular morphology was 90.0% in the persistence subgroup, drastically higher than the 14.3% in the regression subgroup (*P* = 0.001). This finding strongly suggests that even among patients already identified as high-risk by size alone, saccular morphology may signify a more recalcitrant aneurysm subtype, potentially associated with more severe local wall injury, warranting particularly intensive monitoring and management. Given the limited sample size of this subgroup, these findings are hypothesis-generating due to the limited sample size of this subgroup and require validation in larger, dedicated cohorts of patients with large coronary aneurysms. This focused analysis within the highest-risk patients underscores that our anatomy-based CAC model has particular clinical relevance for the patients in whom accurate prognostication matters most.

A key finding of this study is the identification of saccular morphology as a strong risk factor for persistence, which was significant in both univariable and multivariable analyses. Although the multivariable effect estimate was imprecise, the strength of the association (aOR = 7.648) and its striking prevalence in the high-risk persistence subgroup underscore its potential clinical importance. This unfavorable hemodynamic environment has been shown to impair endothelial function, promote inflammatory mediator accumulation, and increase thrombotic risk (5, 6). Therefore, saccular morphology can serve as an intuitive imaging biomarker reflecting more severe local vascular wall injury and a less conducive microenvironment for healing, which may explain its greater resistance to regression (14).

Beyond morphology, the location of the aneurysm also provided critical prognostic information. Furthermore, our analysis confirms that involvement of the left anterior descending coronary artery (LAD) is an independent predictor of aneurysm persistence (aOR = 4.304, *P* < 0.05). This finding consolidates existing evidence from the large-scale RAISE study (11) and reinforces that aneurysm location provides critical prognostic information beyond size alone. Involvement of the LAD, the most critical coronary vessel, may indicate more extensive and severe vasculitis, coupled with higher hemodynamic stress due to the large myocardial territory it supplies, all potentially unfavorable for aneurysm regression (15), corroborating our findings.

The comparative analysis of prediction models provided compelling evidence for a comprehensive anatomical assessment. The significant improvement in discrimination achieved by the full CAC model, coupled with its excellent calibration and superior net benefit on decision curve analysis, underscores its clinical value. This model is particularly useful for identifying patients with moderate-sized aneurysms that harbor additional risk features, thereby enabling more tailored follow-up strategies.

In addition to aneurysm morphology and location, systemic factors such as IVIG resistance and adjunctive immunomodulatory therapy are established predictors of CAA outcomes. In our cohort, univariate analysis of a comprehensive set of baseline variables (Supplementary Table S1) identified several factors associated with one-year persistence, including delayed IVIG treatment, longer total fever duration, and higher platelet count. However, it is noteworthy that IVIG resistance itself did not reach statistical significance in our dataset. This lack of observed association may partly reflect the limited statistical power of our study for certain variables or might suggest that the intrinsic anatomical characteristics of the aneurysm itself play a predominant role in determining its ultimate fate. Therefore, our findings robustly highlight that the aneurysm phenotype provides strong and complementary anatomy-based risk information. They should not be construed as diminishing the established importance of systemic host factors but rather underscore the multifactorial nature of CAA persistence and establish the physical characteristics of the aneurysm as a decisive component within this complex process.

Finally, the strong association of saccular morphology with persistence in the high-risk subgroup (ZM ≥ 5.0) is compelling but requires validation in larger, prospective cohorts due to the small sample size and exploratory nature of that analysis.

Several limitations must be acknowledged. The single-center, retrospective design means that the reported model performance is likely optimistic and requires external validation. The classification of aneurysm morphology, despite being strengthened by a rigorous adjudication process, remains susceptible to a degree of subjective interpretation. Angiography or coronary CTA provides a more definitive assessment of aneurysm morphology (16); however, these modalities are not routinely indicated in the acute phase, and their use at a later stage would not accurately reflect the initial aneurysm phenotype we sought to evaluate. Future studies utilizing these gold-standard modalities are warranted to confirm our findings. Finally, the findings from our high-risk subgroup (ZM ≥ 5.0) analysis, while insightful, are exploratory due to the limited sample size and require validation in larger cohorts. Additionally, this study did not incorporate other potential prognostic factors, such as medication adherence or serial inflammatory markers, whose integration could further enhance future models.

## 5 Conclusion

In conclusion, this study establishes that the intrinsic anatomical characteristics of a coronary aneurysm—its size and location—provide powerful, independent prognostic information. Furthermore, saccular morphology emerged as a potent risk signal, with a strong association to persistence that was particularly salient in the highest-risk patients. These findings support the use of the Comprehensive Aneurysm Characteristic (CAC) model for refined prognostication and highlight the potential critical importance of integrating these anatomy-based assessments into risk stratification paradigms. The findings of this study should be validated in larger, prospective cohorts.

## Data Availability

The original contributions presented in the study are included in the article/Supplementary Material, further inquiries can be directed to the corresponding author.
